# A review of ICT-enabled learning for schoolgirls in Asia and its impacts on education equity

**DOI:** 10.1007/s11423-022-10178-w

**Published:** 2022-12-13

**Authors:** Min Liang, Cher Ping Lim, Jonghwi Park, Norman B. Mendoza

**Affiliations:** 1 Department of Curriculum and Instruction, Faculty of Education and Human Development, The Education University of Hong Kong, Hong Kong SAR, China; 2 United Nations University Institute for the Advanced Study of Sustainability, Tokyo, Japan

**Keywords:** ICT-enabled learning, Girls, Education inclusiveness, Education equity, Asia

## Abstract

The education pathways and opportunities of schoolgirls in Asia are facing different challenges. The empirical studies have implemented Information and Communication Technology-enabled learning to expand such pathways and opportunities and promote education inclusiveness and equity. Through the Gender Analysis Framework, this review paper focuses on exploring and discussing how ICT-enabled learning may expand schoolgirls’ education pathways and opportunities in Asia for inclusive and equitable education. This review covers 30 studies that adopted ICT-enabled learning, synthesizes, and presents four key ICT-enabled learning approaches: Emerging technologies-enabled learning, Digital game-based learning, Mobile-enabled learning, and Computer-assisted learning. Our result discusses about how different approaches in this review (in)directly impact on schoolgirls’ access to assets, their practice and participation in learning activities, belief and perception of their own and other stakeholders’, and how policies accommodate these approaches. The review further suggests several guidelines to develop an inclusive learning environment enabled by ICT to education pathways and opportunities of schoolgirls, thereby enhancing education inclusiveness and equity.

## Introduction

The bedrock of strong economies and resilient society is education equity. It aims to ensure that expected learning outcomes and student learning needs are met in a fair and inclusive manner. That is, besides ensuring that performing students complete their education, expanding pathways and opportunities for members of the marginalized communities is necessary (OECD, 2021). However, several factors such as gender, socioeconomic status, and geography frequently impede such pathways of education inclusiveness and equity. Among these factors, “gender has also remained equally significant over time… the persistent importance of gender for the furthest behind groups indicates that the cultural, institutional, and social context has not changed sufficiently to erase gender divides in education completion over the past decade in Asia-Pacific countries” (UNESCAP, 2019, p.30).

Gender in school is a particular concern for schoolgirls. The inherent barriers that have been placed on schoolgirls’ learning and skill development have primarily been linked to access. Despite significant progress over recent decades in girls’ access to education, girls, particularly those with disadvantaged backgrounds, still lag behind in education (UNESCO, 2018). The barriers to schoolgirls’ learning also exist in the perceived gender stereotypes by educators, families or students themselves. Girls may be considered better at housework, while boys may be considered good at sports. Such gender stereotypes may also reinforce messages that affect girls’ ambitions and perceptions of their roles in society (The World Bank, 2022). Moreover, culturally constructed norms influence schoolgirls’ learning. For instance, in 2018, one in nine girls are married before they turn eighteen years old in Indonesia and girls who marry before eighteen are four times more likely to drop out or not complete secondary education (UNICEF & PUSKAPA, 2020). In addition, policies of the government or schools may affect education inclusiveness and equity concerning schoolgirls. An education policy that emphasizes student-oriented teaching practices benefits girls’ learning gain, whereas those that strictly implement grade retention may favor boys (Hermann & Kopasz, 2021).

To expand schoolgirls’ pathways and opportunities that contribute to education inclusiveness and equity, schools, policymakers, and educators have turned to information and communication technologies (ICT) tools that address education equity in different contexts. ICT in education has the potential to balance the educational disparities for both boys and girls. To realize such a potential, ICT and its relevant tools have to be implemented and adapted to enable better learning design and achieve the desired learning outcomes (Tay, 2016). ICT-enabled learning should use ICT tools that support students’ different learning needs, social contexts, and gender differences that impact students’ learning engagement and outcomes (Dorji et al., 2015). With thoughtful consideration of and intentional focus on gender, ICT-enabled learning may enhance education inclusiveness and equity.

However, few empirical studies exploring ICT-enabled learning in school education have differentiated outcomes by gender (Tauson & Stannard, 2018). Although, reviews have been conducted to examine empirical studies on gender in ICT use and skill development (Qazi et al., 2021), gender equality and ICT in education (Prendes-Espinosa et al., 2020), or ICT in developing countries (Rodriguez-Segura, 2020), few have reviewed studies that cover regional contexts or specific stages of education. These may limit the ability to maximize impactful insights for a deep understanding of certain groups of students in regional contexts. For instance, reviews on studies that implemented ICT-enabled learning in schoolgirls in Asia are scarce. Schoolgirls in Asia are most likely to be affected by a lack of education inclusiveness and equity. This is exacerbated by the COVID-19 pandemic that may have placed schoolgirls at higher risk of not continuing or completing their education. There is an increasing likelihood that girls from larger and poorer families in Southern Asia may not return to school after the pandemic, which puts them at risk of child marriage and early pregnancy (Save the Children, 2020; UNESCO, n.d.^2022^). Without deeper insights into schoolgirls’ patterns of ICT access and its use for education, Tyers-Chowdhury et al. (2021) suggest that it is difficult to build meaningful ways and actions to enhance education inclusiveness and equity.

Schoolgirls’ pathways and opportunities for education face challenges in access, participation, social norms, and educational policies. A holistic approach covering these inter-connected aspects may provide a more thorough analysis and understanding of how ICT-enabled learning for enhancing education inclusiveness and equity. This review thus adopts the Gender Analysis Framework (GAF; Jhpiego, 2020) as a lens to systematically review and examine the empirical studies on ICT-enabled learning. The GAF offers a mechanism to organize data on gender differences in a variety of social contexts and investigate how these differences impact the lives, education and health of men, women, boys, and girls. In this case, GAF enables this review to investigate how ICT-enabled learning may affect girls’ schooling. The GAF includes four dimensions: (1) *access to assets* that include tools and intangible assets such as education, knowledge and information; (2) *practice and participation* concerning different roles and the capacity of a person to participate in economic, political, social activities, and decision-making; (3) *belief and perception* for the cultural norm or belief system that impact the access, roles and capacity; and (4) *institutions, laws and policies* that include the formal and informal rights or rules governing institutions and affecting the access and practice.

The four dimensions of GAF influence and are influenced by the power that affects different levels of decision-making and determines how people are treated and the opportunities provided in education and our society (Jhpiego, 2020). As such, GAF provides a multidimensional framework that systematically examines how ICT-enabled learning influences schoolgirls, thereby aiding the review process and interpretation of the findings of the reviewed studies. To better fit the scope of this review, we narrow down GAF to primary and secondary education (see Methods section) for reviewing the alignment of the empirical studies with the four dimensions of GAF. Therefore, this review examines how ICT expands primary and secondary schoolgirls’ pathways and opportunities for enhancing education equity. The following two research questions are generated.

RQ1: What are the ICT-enabled learning approaches used in the empirical studies for schoolgirls’ learning in Asia?
RQ2: Based on the GAF, how have these ICT-enabled learning approaches expanded pathways and opportunities and enhanced education equity of schoolgirls in Asia?

The key findings of this review are first presented and discussed, followed by our suggestions for gender-inclusive ICT-enabled learning to expand the pathways and opportunities for schoolgirls and enhance education inclusiveness and equity.

## Methods

This review went through three main phases. Phase one involved creating a review protocol and identifying relevant literature based on the four dimensions of GAF. The collected literature was assessed in phase two to see how each paper connected to the RQs. Phase three entailed synthesizing the literature and developing themes based on the RQs. All the authors of this paper were involved in all three phases of the review.

### Phase 1: develop the review protocol and identify the review criteria

We first defined the key terms for developing our review protocol and related parameters based on the RQs. “Asia” in this review refers to countries and territories in the geographical regions of Central Asia, East Asia, South Asia, Southeast Asia, and West Asia, which are categorized for statistical use and publications of the United Nations (UN, 2022; UNSD, n.d. 2022). “ICT” in this review are technologies used for online or offline teaching and learning activities. They include a range of high-end and low-end hardware and software that require different Internet bandwidths or no Internet. “Schoolgirls” or “girls” are female students in primary and/or secondary schools. After these definitions were operationalized in this review, we developed a review protocol (Lasserson et al., 2022) to identify relevant literature and set criteria for all reviewers, as detailed in Table 1 below.

**Table 1:** Review protocol parameters

Databases	Education Resources Information Centre (ERIC), JSTOR Archive Complimentary Collection, Scopus and Web of Science
Article type	Journal articles, conference proceedings, reports
Search on	Title, abstract and keywords for Boolean search terms: (“ict-enabled” OR “ict-enhanced” OR “information communication and technology” OR “education technology” OR “education technologies” OR “digital learning” OR “online learning” OR “ict” OR “computer” OR “internet”) AND (“school”) AND (“schoolgirl” OR “girl” OR “adolescent girl” OR “female student”) NOT (“refugee” OR “Africa” OR “USA” OR “Europe” OR “Australia” OR “America”)
Language	English
Period of publication	Year 2010 to 2021

Consequently, we identified the inclusion and exclusion criteria in Table 2 to validate the review protocol and ensure the relevance of the literature used in this review before going through further analysis. Such criteria could also reduce the impact of reviewers’ biases and promote transparency of review processes (Lasserson et al., 2022).

**Table 2:** Inclusion and exclusion criteria for the review

Inclusion	Exclusion
Must report on empirical ICT-enabled learning (within or beyond school hours)	Meta-analysis or review papers; Opinion or editorial articles
Must report the number or percentage of girls in the study, OR at least show results related to girls	No analysis or results related to the gender difference or girls
Must target students and particularly girls in primary or secondary schools	Early childhood; university students; marginalized groups
Must show measurable outcomes	Only mention the ICT-enabled learning approach and the implementation
Must be conducted in Asia (based on the definition used on this review)	Countries or territories beyond Asia

### Phase 2: conduct the review

**Fig. 1:**
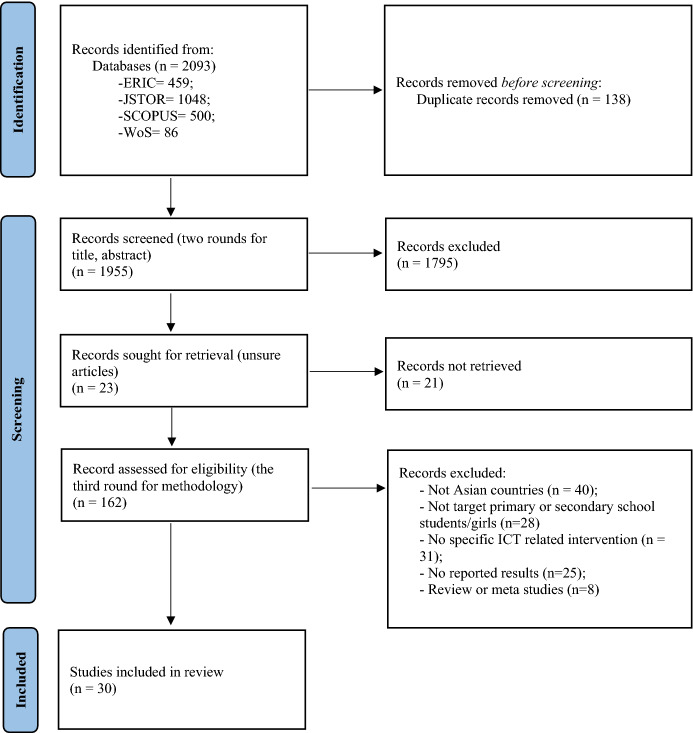
The flow of the searching and screening process

The individual searches of four databases were conducted in September 2021. Before searching and screening, we discussed and agreed upon the aim and RQs of the review and the review protocol. The search result from four databases was 2093 in total. After removing 138 duplicated articles, the results went down to 1955. Then each reviewer was responsible for one or two databases for the first round that involved—title screening (i.e., considering the relevance to the RQs from the titles of the literature). In every round, reviewers labelled the articles as “unsure” if they could not decide by themselves. To ensure each article undergoes blind review by at least two reviewers, the result of title screening (including the “unsure” articles) was mixed and re-distributed among reviewers for the second round that involved abstract screening. These two rounds screened out 1795 articles and narrowed down the review to 162 screened articles and 23 “unsure” articles. These were moved to the third round—methodology screening. In the methodology screening, reviewers were asked to thoroughly verify the methodology section of the articles by ensuring that the target participants or intervention aligned with the inclusion and exclusion criteria of the review. The reference lists of screened articles were also checked for more potentially related articles using the snowball method (White, 2009). After the third round, all the included articles were collected by the first author to conduct individual screening again and confirm with other reviewers to ensure the quality. In the end, 30 articles were deemed eligible for further analysis, and the screening was completed in early October 2021. Figure 1 shows the flow of searching and screening articles, the number of articles we have identified, included and excluded, and the reasons for exclusions.

### Phase 3: synthesize the included studies

This phase involved data extraction from the included studies and further analysis of their findings based on the GAF. In this review, the four dimensions of GAF were applied in the context of primary and secondary education. *Access to assets* in this review refers to schoolgirls’ access to necessary resources and knowledge for their learning and growth (e.g., access to ICT tools or skills). *Practice and participation* refer to schoolgirls’ engagement and role in the school activities and beyond (e.g., the engagement in a game-based learning activity). *Belief and perception* refer to the perspectives or stereotypes of schoolgirls’ learning and capacity (e.g., girls may be assumed as not good at computational thinking). And *policies* refer to the rights or rules of schools or government that could influence the three dimensions mentioned above (e.g., a national policy that requires all students to attend basic ICT courses). These four dimensions are interrelated, and one dimension could influence another and vice versa. They have an impact on and are affected by the power that determines how schoolgirls are treated and given access to opportunities for education at various levels of decision-making.

The analysis of the findings of the included studies followed Wolfswinkel et al. (2013) three steps of rigorously analyzing literature: open coding, axial coding, and selective coding. Guiding by the RQs through GAF, reviewers narrowed down the codes emerging from the findings step by step. In the open coding, the reviewers read the findings of each study in detail and generated a series of codes for two RQs (e.g., ‘mobile phone’ or ‘mobile applications (apps)’ for RQ1; and ‘engagement’ and/ or ‘teachers’ for RQ2). Some studies were labelled with several codes for each RQ in this step. In the axial coding, codes assigned in the last step were categorized to capture groups of concepts (e.g., ‘mobile phone’ and ‘mobile apps’ were combined under ‘mobile’). In this step, codes under RQ1 for each study would be further examined, and then one code representing the key usage of ICT in that study would be kept. For instance, we kept ‘game’ for Jain and Yammiyavar’s (2015) study because it mainly examined a digital game for girls’ learning, despite being deployed on a tablet. Given that one study may align with more than one GAF dimension, codes under RQ2 were permitted to be in more than one category of codes (e.g., ‘participation’, ‘perception’, ‘peer discussion’ for Jain & Yammiyavar’s study) to generate themes. In this process, re-reading the articles, re-examining and refining the codes were conducted accompanying with continual discussions among team members. The categories of codes were then finally merged into four major ICT-enabled learning techniques for RQ1 and four major key themes for RQ2 (e.g., ‘Tablet’, ‘iPad’ and ‘mobile phone’ were included in ‘mobile-enabled learning’, ‘engagement’ and ‘experience’ were further combined as ‘participation’). The whole process was completed in early January 2022.

## Key findings and discussions

This review has 30 publications that span 13 Asian countries and territories. Most of them were conducted in East Asia (15 studies) and West Asia (11 studies), followed by studies in South Asia (2 studies) and Southeast Asia (2 studies). Seventy percent targeted both boys and girls (21 studies), and 30% solely targeted girls (9 studies). As for the length of the studies, 83% of them were finished within months or in one semester (25 studies), while around 17% were conducted for at least one school year (5 studies). Quantitative methods were adopted in 63% of the studies (19 studies), and mixed methods that supplement questionnaires with interview data were also adopted in about one-third of the studies (11 studies).

The key findings and discussions are organized based on the RQs. Each key findings and discussions detail ICT-enabled learning approaches used for schoolgirls’ learning in Asia and how they align with the GAF to expand the pathways and opportunities for schoolgirls, and thus enhance education inclusiveness and equity.

### ICT-enabled learning approaches used for schoolgirls’ learning in Asia

With respect to RQ1, four major ICT-enabled learning approaches were identified from the reviewed studies. These approaches are not mutually exclusive, but each of them adopts a major ICT tool for teaching and learning. These inter-related approaches are: (1) Emerging technologies-enabled learning (3 studies); (2) Digital game-based learning (6 studies); (3) Mobile-enabled learning (7 studies); and (4) Computer-assisted learning (14 studies).

The *emerging technologies-enabled learning* uses comparatively high-end ICT tools, such as Augmented Reality (AR)/Virtual Reality (VR), 3D printing, robots, etc. They may provide authentic and rich experiences to students and better interactions between students and the learning materials. They also require more investment from schools and/or governments on the required devices and stable Internet with higher bandwidth. Two of the three studies adopt this approach in developed economies (e.g., United Arab Emirates, South Korea), where stable and high internet bandwidth may be more accessible.

*Digital game-based learning (DGBL)* mainly features either existing or in-house produced digital games that may require a high or low internet connection bandwidth and various devices (e.g., computer, tablet, smartphone). For teaching and learning, one particular feature of DGBL—single or multiple player mode - that is different from other approaches may enable more collaboration, cooperation and discussion among students. In this review, it was found that schoolgirls in both developing and developed economies could access to DGBL for educational purposes.

*Mobile-enabled learning* mainly relies on mobile devices such as tablets, mobile phones, and apps to conduct learning activities and requires different internet connection bandwidths. In this review, the approach covers iPad, education tablets, exiting mobile apps such as WhatsApp and a tailor-make eBook learning system. It is also found that this approach is adopted for learning activities in both developing and developed economies.

*Computer-assisted learning (CAL)* in this review refers to those who mainly rely on desktop or laptop computers with other ICT tools to enhance teaching and learning, while excluding the features of the previous three types (i.e., emerging technologies, digital games and mobile). CAL in this review has different manifestations for teaching and learning: computers are used to support other hardware such as interactive whiteboard (Chen et al., 2020); computers to support existing software such as mBlock (Gunbatar & Karalar, 2018); computers to support ICT training program or remedial tutoring (Mo et al., 2015); computers to support multimedia resources (Wang et al., 2015). It is found that CAL is the most common approach among the included studies in both developing and developed economies.

Four ICT-enabled learning approaches require different internet bandwidth due to different contexts of use. They may require lower or even no bandwidth if the required devices are preloaded with educational resources. They were used in-class or out-of-school interactions and activities. There are two prominent roles of ICT tools in such approaches for teaching and learning in this review. One is to supplement or substitute conventional educational resources for students to learn subject knowledge (Ali et al., 2019; Tsai, 2017), and the other is to function as a major tool for improving students’ subject knowledge (Mo et al., 2015; Tho et al., 2015;), or developing students’ particular skills (Hsieh & Chen, 2019; Jeng & Liu, 2016). Despite being used widely, the two roles of ICT tools in ICT-enabled learning may have not yet been explicitly examined, particularly in Asia or concerning girls’ learning. This review could help Asian countries better understand how ICT tools can be integrated into interventions to improve education inclusiveness and equity concerning gender and local contexts. The key information of the included studies in this review is summarized in the Appendix.

### ICT-enabled learning and schoolgirls’ learning inclusiveness and equity

**Fig. 2:**
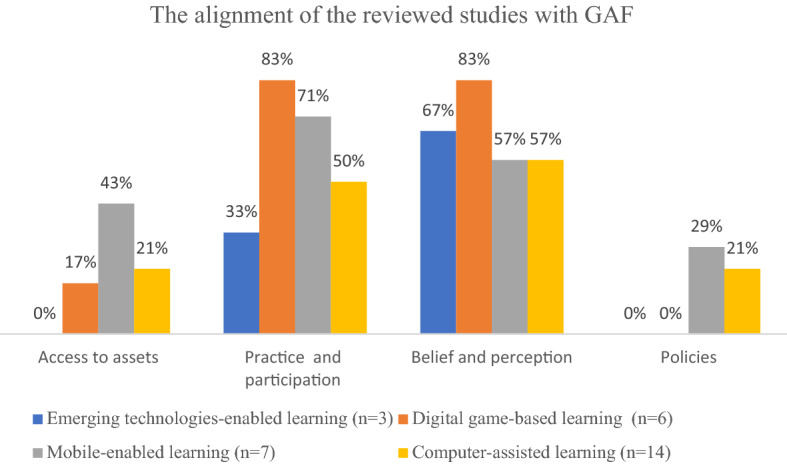
The alignment of the reviewed studies with GAF

For answering RQ2, which focuses on how ICT-enabled learning may expand the pathways and opportunities for schoolgirls’ learning, we summarized the alignment of the reviewed studies with GAF in Table 3 and visualized it in Fig. 2 below before eliciting and discussing themes in this section.

**Table 3:** The alignment of the reviewed studies with GAF.

	Access to assets	Practice and participation	Belief and perception	Policies
Emerging technologies-enabled learning (n = 3)	0	1 (33%)	2 (67%)	0
Digital game-based learning (DGBL) (n = 6)	1 (17%)	5 (83%)	5 (83%)	0
Mobile-enabled learning (n = 7)	3 (43%)	5 (71%)	4 (57%)	2 (29%)
Computer-assisted learning (CAL)(n = 14)	3 (21%)	7 (50%)	8 (57%)	3 (21%)
Total (n = 30)	7 (23%)	18 (60%)	19 (63%)	5 (17%)

It is found that the reviewed studies have aligned with the GAF across different ICT-enabled learning approaches, and one study may align with multiple dimensions of GAF. There are around 23% (7 studies) of the studies aligned with *access to assets*, 60% (18 studies) aligned with *practice and participation*, about 63% (19 studies) aligned with *belief and perception*, and around 17% (5 studies) aligned with *policies*.

When comparing different approaches in the reviewed studies, it is found that mobile-enabled learning (43%) leads to more *access to assets* in this review, followed by CAL (21%) and DGBL (17%); DGBL (83%) leads to more *practice and participation*, than mobile-enabled learning (71%), CAL (50%), emerging technologies-enabled learning (33%); DGBL (83%) also leads to more changes of *belief and perception*, followed by emerging technologies-enabled learning (67%), mobile-enabled learning (57%) and CAL (57%); As for *policies*, only mobile-enabled learning (29%) and CAL (21%) reflect the government or school policies.

From the alignment results, four themes were identified from the reviewed studies. Specifically, namely, (1) ICT-enabled learning improves schoolgirls’ access to resources, knowledge, and skills; (2) ICT-enabled learning improves schoolgirls’ practice and participation in learning; (3) ICT-enabled learning may change beliefs and perceptions about girls; (4) Policies of ICT-enabled learning accommodate education inclusiveness. The themes highlight the details of key ICT-enabled learning approaches utilized in the reviewed studies. In addition to presenting the themes below, this section reveals that teachers’ roles and the stakeholders’ support may impact the alignment with GAF.

#### ICT-enabled learning improves schoolgirls’ access to resources, knowledge, and skills

Different ICT-enabled learning approaches have been found to support schoolgirls’ access to resources, knowledge and skills. In this review, mobile-enabled learning and CAL expanded girls’ access to resources, knowledge and skills. It may be because mobile devices are more common ICT tools that a household owns and affords to support schoolgirls’ access to learning opportunities. For instance, mobile phones and apps (e.g., Facebook) are found popular among urban adolescent girls in India to access various knowledge and information (Singh & Jain, 2017). When supported by the government, tablet computers in teaching and learning make information and resources more accessible and bring the same technology experience and enjoyment to boys and girls in underdeveloped rural areas (Pruet et al., 2016). Mobile apps on mobile devices such as iPad have become prevalent, and schoolgirls with different abilities thus can extend their learning beyond classes and enhance their self-regulated and collaborative learning in developed economies (Al-Shaya & Oyaid, 2021; Tay, 2016). CAL used for in-class teaching and learning, and out-of-class tutoring also enables girls to access computers and more information about subject knowledge (Yang et al., 2013), develop scientific inquiry skills in a virtual environment and promotes learning science among girls (Wang et al., 2015).

Girls can be introduced to another engaging and interactive method of accessing knowledge and information by using DGBL in teaching and learning. When a traditional Indian game was redesigned in a digital format, rural girls who were previously reluctant to disclose health information became more open to discussion with peers (Jain & Yammiyavar, 2015). However, learning made possible by emerging technologies might not directly improve girls’ access. Despite being given a chance to try emerging technologies that are rare in schoolgirls’ personal lives as a novel way to learn a subject matter, schoolgirls still have less access to the Internet at home than boys do (Ali et al., 2019). Additionally, if schools primarily use traditional teaching methods, cultural beliefs that can get in the way of schoolgirls’ learning may continue to restrict their access to learning (Aljaber, 2018; Al-Shaya & Oyaid, 2021).

#### ICT-enabled learning improves schoolgirls’ practice and participation in learning

In this review, girls’ improved participation and practice are observed in more than half of the included studies that have featured DGBL, mobile-enabled learning and CAL. Emerging technologies such as AR incorporated in teaching materials motivate girls to create better stories in their own way (Yilmaz & Goktas, 2018). Digital games connect students to course content in teaching and learning contexts inside and outside of the classroom. As students play digital games, they collaborate or cooperate with peers to further their grasp of the course content. Studies show that girls scored better than boys in engagement and learning outcomes when DGBL was implemented to impact students’ engagement and deeper comprehension of course content (Khan et al., 2017; Yang & Quadir, 2018; Tsai, 2017).

Mobile-enabled learning that either incorporates iPad and/or relevant apps was found to better engage girls’ in and beyond class hours. Tay (2016) ’s longitudinal study found that the iPad was associated with more engagement and interaction among girls with different learning abilities. When guided by the appropriately selected mobile apps, mobile-enabled learning may better facilitate girls’ language learning in English classes than conventional teaching methods (Al-Bogami & Elyas, 2020) or attract girls to participate in more physical activities during weekends (Seah & Koh, 2021).

The addition of computer-assisted multimedia to texts and images for teaching and learning helps students pay attention and participate more effectively, which helps them comprehend basic subject knowledge. Such interaction with computer-assisted multimedia demonstrates girls’ comparable improvement to their male classmates (Nusir et al., 2013). Computer-mediated discussion platforms with online teachers’ guidance have motivated girls’ online presence, and peer-led interaction helps them co-construct more quality reasoned arguments and collective argument chains than boys (Asterhan et al., 2012). For science inquiry-based projects in school, the computer-mediated platform such as wiki could also extend girls’ online engagement to more meaningful offline interactions for their learning (Lau et al., 2017).

Such improved participation and practice can lead to girls’ better cognitive performance and social relationships (Hsieh & Chen, 2019), better academic performance and learning outcomes (Al-Bogami & Elyas, 2020; Khan et al., 2017; Mo et al., 2015; Tho et al., 2015; Tsai, 2017; Wang et al., 2015).

However, students’ knowledge foundation may influence their performance in these studies. For example, girls who have repeated playing and answering quizzes or have better subject knowledge than boys before participating in activities probably would gain higher scores (Tsai, 2017). To further improve girls’ participation in ICT-enabled learning activities, educators can be informed about the gender difference for better pedagogical design to enhance girls’ learning motivation and equally motivate both boys and girls (Yang & Quadir, 2018). For example, boys and girls may have different gaming flow experiences in DGBL (Yang & Quadir, 2018); doing team projects may be more suitable for girls (Noh & Lee, 2020), or gender-balanced student groups may enable better collaboration between boys and girls (Baek & Touati, 2020). Also, educators can also adopt low-cost devices for ICT-enabled learning activities to cover more girls to participate and enjoy subjects such as physics (Tho et al., 2015).

#### ICT-enabled learning may change beliefs and perceptions about girls

Improved participation and performance may increase girls’ interest in and motivation to use ICT to support their own learning. Such increase in interest and motivation we found in most studies incorporated four different ICT-enabled learning approaches in this review may stem from the changes in their perceptions of ICT and in girls’ capacity.

After trying emerging technologies such as 3D printing in teaching and learning, schoolgirls’ interest and enjoyment in using ICT tools were observed, and their perceived value of ICT was improved (Ali et al., 2019). Using robots to learn programming helped the researchers to see that both boys’ and girls’ computational thinking and creativity could be improved in general since the gender difference in such improvement was not statistically significant (Noh & Lee, 2020). Experiencing DGBL that incorporated with subject knowledge or in learning tasks, girls from primary school (Yang & Quadir, 2018), high school (Tsai, 2017) or rural areas (Jain & Yammiyavar, 2015) showed a positive attitude and motivation toward DGBL, or their preference in cooperative tasks (Baek & Touati, 2020), and their confidence on DGBL (Khan et al., 2017). For example, introducing a game-based achievement system into English learning activities helped researchers to see girls’ and boys’ different gaming flow experiences, which can predict their learning motivation in DGBL (Yang & Quadir, 2018).

Mobile-enabled learning incorporates mobile devices and appropriately chosen educational apps for learning activities to enable girls to develop a positive attitude toward and see ICT’s value in their education (Al-Bogami & Elyas, 2020; Pruet et al., 2016). When mobile devices enabled students to interact in and out of the classroom, Aktas and Çan (2019) observed that girls’ self-efficacy, which is the belief in one’s capacity to accomplish a task (Zimmerman, 2000), was increased. Additionally, when mobile-enabled learning was used on tablets with an eBook learning system for in-class instruction, researchers and educators found that boys and girls have different reading habits. Such gender differences in reading habits may help schools and educators rethink the design and implementation of mobile-enabled learning in classrooms to cater for both genders with equity (Huang et al., 2013). As for studies that incorporated CAL, ICT training or remedial tutoring programs were found to benefit boys and girls equally (Yang et al., 2013) and changes their perception of ICT self-efficacy and attitudes toward ICT usage for learning (Barak & Asad, 2012; Lau et al., 2017; Tam et al., 2020).

The studies reveal to the educators and researchers that girls’ learning gain could be equal to boys’, while their learning style or experience may be different from boys’. For example, girls may have different gaming experiences and motivations (Barak & Asad, 2012; Yang & Quadir, 2018), spatial abilities (Jeng & Liu, 2016), enjoyment and improvement in science performance (Tho et al., 2015). Girls may use different reading strategies from boys to understand the content of eBooks (Huang et al., 2013), and they may have a higher visual learning style than boys when using laptops (Pruet et al., 2016). When some of the differences are associated with girls’ ICT self-efficacy, perceived value and difficulties in using ICT, they may be reduced by granting more ICT-enabled learning opportunities to schoolgirls (Tam et al., 2020). Besides, such opportunities can pay attention to enhancing girls’ ICT self-efficacy and reducing perceived difficulties in using ICT, eventually promoting their development and alleviating gender stereotypes in certain subjects.

#### Policies of ICT-enabled learning accommodate education inclusiveness

In this review, comparatively fewer studies align with the policy dimension of GAF. Although the number of studies is relatively small (5 studies which incorporated mobile-enabled learning and CAL), we consider that they still help us to understand how policies could accommodate education inclusiveness and equity at the national or school level.

When mobile-enabled learning was found to increase girls’ access to education in Saudi Arabia, which has implemented *Saudi Vision 2030* that advocates the necessity of change and women empowerment (Al-Shaya & Oyaid, 2021), it may encourage the national government to further invest resources to ICT-enabled learning for improving girls’ learning opportunities. Pruet et al. (2016) study in Thailand, which has implemented *One Tablet PC Per Child* initiative nationwide, found that girls who used tablets for learning showed the same positive attitude and technology experience as boys but with different learning styles. However, students from rural areas faced higher learning competitiveness and higher levels of anxiety about tablet use than those from urban areas. Such findings may alert the national government and schools to pay attention to the differences of gender and geographical location to further improve the effectiveness of the initiative.

In China, the 12th five-year plan for integrating ICT into education has been launched for building computer rooms in rural schools by 2020 (Mo et al., 2015). CAL programs such as *One Laptop Per Child* providing remedial tutoring were found to benefit rural primary girls and boys in China equally (Yang et al., 2013), and girls in lower grade levels improved more than boys in math (Mo et al., 2015). To sustain girls’ short-term improvement brought by CAL, national or local governments must manage and sustain ongoing efforts to provide computers and necessary ICT tools to related schools (Mo et al., 2015; Yang et al., 2013). However, policies may not improve education inclusiveness if they pay little attention to gender differences. For example, Meishar-Tal and Shonfeld (2019)’s study found that in a school that adopted a *paperless classroom* policy that uses laptops and other ICT tools to replace printed materials, girls, particularly those with weak academic performance, were found to prefer handwriting on paper and have less willingness to use ICT tools for learning. Moreover, students who had experienced such a policy in this school became less supportive of the use of computers for reading and writing than younger students. In this case, girls’ participation in learning activities may have been deterred, further influencing the school policy’s refinement and/or continuity. Therefore, apart from gaining a better understanding of gender differences in school learning, policy development and implementation by collecting gender aggregated data that reflect the research evidence is pivotal to guiding ICT-enabled learning at the national, district and school levels.

In summary, ICT-enabled learning in this review aligns with GAF to different extents. First, ICT-enabled learning provides and improves schoolgirls’ *access* to ICT tools, information, and skills. The improved access enables them to interact with subject content more frequently and communicate with teachers and classmates outside the classroom. Second, ICT-enabled learning increases girls’ *practice and participation* in learning activities in and out of school. Such increased participation can lead to girls’ better academic performance, cognitive skills development, and collaboration in school subjects. Third, with increased access and participation, the *belief and perception* of girls’ capacity, the usefulness of ICT, and educators’ perception of gender differences in learning may change. However, fewer studies reflect the *policies* that guide the previous three dimensions in this review. This variation may be due to empirical studies usually conducted by individuals or a small team of researchers targeting a specific phenomenon in a particular context. Such empirical studies provide gender-segregated data and research evidence for government and schools to develop and implement policies of ICT-enabled learning to enhance education inclusiveness.

Besides the abovementioned four themes that are informed by the four dimensions of GAF, we also noted teachers’ roles and the stakeholders’ support might impact girls’ ICT-enabled learning. Teachers were found to have a role or participated in around 70% of the included studies. For example, they helped supervise and observe students’ performance and difficulties (Mo et al., 2015) and offered feedback on the studies (Khan et al., 2017; Tho et al., 2015). Or they worked closely with the researchers to design lesson plans for ICT-enabled learning adopted in the studies and to observe students’ performance (Barak & Asad, 2012). Teachers’ role and participation in these studies appear to be essential in guiding girls’ better participation and collaboration based on teachers’ improved understanding of the gender differences in ICT-enabled learning activities (Asterhan et al., 2012; Lau et al., 2017). Such understanding may enable frontline teachers to use or adjust ICT-enabled learning to better support girls in their daily learning activities. Such requires researchers to conduct briefings and training for teachers involved. Teachers thus can get the basic knowledge of ICT tools and ICT-enabled learning, particularly those working in rural schools (Mo et al., 2015). To enable ICT-enabled learning for education inclusiveness in schools, how teachers use ICT tools for teaching and learning is the key (Chen et al., 2020). Therefore, effective and structured professional development in using ICT tools is critical (Chen et al., 2020; Khan et al., 2017) for teachers to understand and better integrate ICT tools with subject content and consider gender differences in schoolgirls’ learning.

Apart from teachers, the stakeholders such as parents and family members also play a critical role in the impact of ICT-enabled learning. As rural Indian girls feel embarrassed or hesitant to participate in DGBL to discuss health information when males are around, boys and other male members in girls’ families should also be educated about puberty while educating girls (Jain & Yammiyavar, 2015). Although girls seem to obtain the same improvement as boys or even better than boys in the ICT-enabled learning activities, girls’ access to the Internet and ICT tools at home is still very limited (Ali et al., 2019). Access to digital devices at home and in schools is significantly associated with girls’ performance of digital citizenship competencies (UNESCO, 2019) and their enjoyment and participation in ICT-enabled learning activities (Hsieh & Chen, 2019). In addition, “utility value intervention” targeted at parents may be helpful to promote their perceived usefulness of ICT and possibly lead to girls’ increased access to the Internet and ICT tools (Ali et al., 2019, p. 148). Hence, support from stakeholders such as parents and family members is required to extend girls’ learning experience out of the classroom and sustain the improvements gained from ICT-enabled learning.

## Guidelines for designing and implementing ICT-enabled learning to empower schoolgirls

In this review, we argue that ICT-enabled learning has the potential to increase education inclusiveness and equity. Its potential stems from the evidence of improving girls’ access to ICT tools and knowledge, enhancing their participation in learning activities and leading to a change in the perception of girls, educators, and related stakeholders. Realizing such potential calls for more guidance from educational policies. In addition, educators and stakeholders, who have the power of decision-making in the classroom, schools, communities, and families, play pivotal roles in implementing ICT-enabled learning to support schoolgirls’ learning. Based on these findings, we discuss four guidelines in this session about how ICT-enabled learning could be designed and implemented in schools to realize its potential to increase girls’ access and participation in learning activities, facilitate changes in belief and perception, as well as inform policies for enhancing education inclusiveness equity.

First, ICT-enabled learning needs to be implemented affordably and cost-effectively to ensure girls’ *access*. Although emerging technologies-enabled learning provides schoolgirls with access to AR or robots and participation in learning activities, it may be limited to a small-scale and a specific venue such as school computer rooms or certain countries or schools. *DGBL* enables students to engage in playful learning in different domains and school subjects (Homer et al., 2020). However, one of the barriers to DGBL adoption in developing economies is the cost of developing and deploying games in local schools, including the required resources and teachers’ capacity for integrating games with course content. Such barriers underscore the need for affordable game design and the specific affordances of games for learning (Lim et al., 2018). Hence, learning activities using various ICT tools can benefit schoolgirls in low-income countries or rural areas. Also, cost-effective and reusable devices are affordable to local governments, schools, and families to adopt for ICT-enabled learning. For instance, Wii-mote is cost-effectively used for conducting activities about learning physics (Tho et al., 2015). “Help Pinky” deployed in the Akash tablet is an affordable ICT tool in rural India (Jain & Yammiyavar, 2015). ICT-enabled learning can be more inclusive, providing more opportunities for girls and potentially enhancing education equity through the informed selection of ICT tools that meet the varied needs of schools and families.

Second, the design of ICT-enabled learning needs to be inclusive and gender-sensitive to improve girls’ *practice and participation* in learning activities. Inclusive design requires designers and educators to be sensitive to the gender differences in learner preferences and capacities and provide materials to maintain girls’ interests (Pruet et al., 2016; Yang & Quadir, 2018). The designers and educators also need to be sensitive to the contexts of use, such as the local culture in the targeted area or community where ICT-enabled learning is implemented. For instance, the digital game “Help Pinky” shows designers’ understanding of local culture and what schoolgirls may feel about puberty-related topics (Jain & Yammiyavar, 2015). It is culturally relevant and developed for educating schoolgirls in their local language Assamese. Therefore, needs and situation analysis can be conducted before designing ICT-enabled learning. Local partners and teachers can be invited to co-designing and developing ICT-enabled learning activities for their acceptance and localization. In addition, women must be included in all aspects and processes of ICT-enabled learning. Such a women’s role can observe the gender issue and suggest the design and implementation of ICT-enabled learning for empowering women and schoolgirls (EQUALS, 2019).

Third, inclusive and gender-sensitive pedagogies facilitate changes in *belief and perception*. This review shows that teachers’ involvement varied from supervision and observation to being given workshop training, collaborating with researchers, and using the ICT tools for ICT-enabled learning activities. Teachers’ involvement helps schools and teachers themselves gain support and buy-in for ICT-enabled learning, and notice gender differences when using ICT tools for teaching and learning. For example, their better understanding of group structure in the ICT-enabled learning activities may optimize girls’ performance and achievement. However, teachers may also harbor gender stereotypes about which students will benefit from ICT (Pitchford et al., 2019), while their interaction with students around educational materials matters most to improve learning (Cohen & Ball, 1999). Hence, gender-sensitive teacher education is needed (UNESCO, 2020), and their professional development should include inclusive and gender-sensitive pedagogies (Webb et al., 2020). With gender-sensitive teacher education and professional development, teachers in teaching and learning activities may raise their awareness, observe the gender difference, and provide gender-sensitive instructions that equally benefit both boys’ and girls’ learning. In addition, they may rethink and even transform their traditional roles to be facilitators of creating a student-centred learning environment and helping girls understand their roles and capacities in our society (Tay, 2016).

Inclusive and gender-sensitive pedagogies training should also extend to girls’ parents and related family members. When broader socio-cultural issues may be one of the reasons that schoolgirls have less interest in ICT or science (Barak & Asad, 2012), the pervasive gender stereotypes in education may be “the sins of the parents” (Eble & Hu, 2019). Some parents may still believe it is unnecessary to teach their daughters how to use computers, despite having computers at home and allowing boys to use them (Meno, 2012). Parents and family members who are vital agents that mediate the relationships among educators, students, and the resources (Ganimian et al., 2020), are not only the gatekeepers to schoolgirls’ access to the Internet and ICT tools outside of school (Webb et al., 2020), but also the direct or indirect influencers of girls’ perception of their own roles and capacity.

Fourth, inclusiveness and gender sensitivity should be embedded in developing and implementing educational *policies*. There are few studies directly aligned with policies in this review. However, a supportive national or regional policy is crucial in guiding ICT-enabled learning and securing girls’ access and participation in education. A meta-analysis of global policies conducted by Borokhovski et al. (2018) reveals that only seven of eighty-seven ICT-focused or gender-focused policy documents are from Asia. Only two of them have particular monitoring measures in place, and none of them focuses on primary and secondary education. Therefore, inclusiveness and gender sensitivity need our urgent actions to collect gender aggregated data, reflect the research evidence in developing policies for ICT-enabled learning, and monitor and evaluate gender aggregated learning gains before scaling up. Despite the increased policies promoting nationwide access to education or ICT, they often fail in practice (UNESCO, 2020). Therefore, decision-making of policies at different levels may refer to the GAF as much as a needs and situation framework and embed the dimensions of access, participation, and perception change in the development and implementation process. In such a process, women should be involved in all aspects to observe the gender issue and suggest ways to empower schoolgirls (EQUALS, 2019). When evaluating the policy implementation, the result should be made available to the policymakers, educators, and parents and reviewed regularly to improve education equity.

In summary, the four guidelines covering the four dimensions of GAF suggest how ICT-enabled learning could be designed and implemented to increase girls’ access and participation in learning activities, facilitate changes in perception and inform policies. However, given that these guidelines are suggested based on reviewing the studies conducted in Asia, putting such guidelines into practice requires educators and policymakers to consider the local contexts.

## Conclusion

This review examines studies that adopted different ICT-enabled learning approaches for schoolgirls’ learning in Asia. Through the lens of GAF, most studies on ICT-enabled education can align with the dimension of access to assets, practice and participation, belief and perception of GAF, but less to the dimension of policies. We suggest four guidelines for inclusive and gender-sensitive design and implementation of ICT-enabled learning that may be able to expand schoolgirls’ pathways and opportunities for education based on the key findings of this review. Given that the digital divide in education extends beyond the issue of technology access (The World Bank, 2020), these suggestions target each dimension of GAF in the context of the education sector. With inclusive and gender-sensitive design and implementation of ICT-enabled learning, different stakeholders, including designers, educators, parents, and policymakers, may better utilize the potential of ICT-enabled learning to expand the pathways and opportunities for schoolgirls’ learning to enhance education inclusiveness and equity.

As for future research, we first suggest that comparatively long-term empirical studies about ICT-enabled learning should be encouraged and supported. In this review, some studies were conducted in a short-term intervention (Ali et al., 2019; Jain & Yammiyavar, 2015). In a short-term intervention, girls’ improved interest and performance may come from short-term interest and excitement in accessing the ICT tools they barely accessed before. When girls are exposed to ICT-enabled learning more frequently, their learning shows better results (Tsai, 2017) and outperforms boys over time (Chen et al., 2020).

Second, schoolgirls in South and Southeast Asia still face difficulties accessing and participating in education (Pappu, 2020). Hence, future research can pay more attention to the potential of ICT-abled learning brought to this group of girls. Besides, further exploration of the roles of teachers and parents in ICT-enabled learning will also bring more insights about designing and implementing ICT-enabled learning in and out of classrooms that can enhance education inclusiveness and equity.

Third, we echo the suggestion of Prendes-Espinosa et al. (2020) to broaden the studies qualitatively. Studies that adopt quantitative methodology provide an overview of gender differences; the reasons behind such a difference also deserve attention by collecting views from girls themselves and related stakeholders. For example, how have girls in rural China engaged in CAL (Mo et al., (2015)? Or what may have impacted girls’ interest in learning image-processing (Barak &Asad, 2012)? Qualitative data can provide more in-depth views for conducting research, teaching and learning in schools and policymaking concerning inclusiveness and gender sensitivity. Views from girls and stakeholders can also reveal the socio-cultural issues behind their access, participation and perception in ICT-enabled learning activities. With quantitative and qualitative data, ICT-enabled learning can be better designed in the local contexts for enhancing education inclusiveness and equity.

Limitations of this review lie in its concentration on the studies conducted in countries and territories in Asia and published in English that could be accessed within a specific timeframe. Some relevant studies from other databases and those published lately have not been covered. This review thus may not be able to represent the entire body of research on ICT-enabled learning around the globe. Besides, this review only focuses on evidences from empirical research without further analyzing context-related elements among these countries and territories such as Gross Domestic Product (GDP), religion, and geographical location that may impact ICT-enabled learning approaches on schoolgirls’ pathways to education. Context-related analysis can be one of the future review approaches.

## Appendix

The key information of the included studies in this review.

**Table:**

Authors and location	Grade/school	Sample size (girls)	Duration	Method	Role of ICT tools in the teaching and learning	Alignment with GAF
(1) Emerging technologies-enabled learning						
Ali et al. (2019) (UAE)	Grade 5, three public schools	276 girls	1 unit	Quantitative	Using 3D printing to learn volcano and hand-on experience to create a volcano	Perception (of the usefulness of ICT)
Noh and Lee (2020); (South Korea)	Grade 5–6,	84 girls out of 155 students	11 weeks	Quantitative	Using a robot in T&L to learn programming for computational skill	Perception (of girls’ computational thinking skills and creativity by a robot)
Yilmaz and Goktas (2017) (Türkiye)	Grade 5, an elementary school	54 girls out of 100 students	4 weeks	Quantitative	Using AR cards to help students to create stories with selected characters and display 3D such characters in their own ways	Participation (in creating stories)
(2) Digital game-based learning						
Baek and Touati (2020) (South Korea)	Grade 6, an elementary school	80 girls out of 140 students	5 weeks	Quantitative	Playing Minecraft to collaborate or cooperate in groups to build and replicate houses	Participation (in DGBL), perception (of girls’ preference for collaborative learning)
Hsieh and Chen (2019) (Taiwan)	Grade 5–6, a primary school	63 girls out of 123 students	10 weeks	Quantitative	Playing Pokémon Go for cognitive performance and emotional intelligence	Participation (in physical activities)
Jain and Yammiyavar (2015) (India)	12–14 years old, rural area	12 girls	2 sessions (no specific duration)	Mixed	A two-player game to enable girls to learn health knowledge and trigger discussion and help out each other	Access (to health information), participation (in discussion), perception (girls’ puberty)
Khan et al. (2017) (Pakistan)	Grade 8, a private school in suburban Pakistan	36 girls of 72 students	3 weeks	Mixed	Using a game to connect with course content in science learning activities	Participation (in learning activities), perception (of girls’ interest and confidence in the game)
Tsai (2017) (Taiwan)	Grade 9, a high school	40 girls out of 74 students	3 weeks	Quantitative	Using a quiz game for energy knowledge acquisition in single or multiple-player mode	Participation (in learning activities), perception (of girls’ attitude to the game)
Yang and Quadir (2018) (Taiwan)	Grade 3, an elementary school	24 girls out of 50 students	3 weeks	Mixed	Digital activities and game-based achievement system in English learning actives	Perception (of girls’ gaming experience and motivation)
(3) Mobile-enabled learning						
Al-Bogami and Elyas (2020) (Saudi Arabia)	A private middle school	20 girls	5 weeks	Mixed	Using iPad apps in classes to facilitate language learning and engage students.	Participation (in EFL activities), perception (of using App for learning)
Al-Shaya and Oyaid (2021) (Saudi Arabia)	A university-affiliated secondary school	35 girls	Not reported	Mixed	e-Learning platform to encourage girls to achieve high-level interaction in an e-Learning environment	Access (to e-Learning), participation (in e-Learning), policy (of government)
Aktas and Can (2019) (Türkiye)	Grade 11, a high school	12 girls of 19 students	8 weeks	Mixed	Using WhatsApp for online discussion outside of school	Perception (and attitude of self-efficacy)
Huang et al. (2013) (Taiwan)	A technology-rich elementary school	81 girls out of 166 students; & 11 out of 34 students	80 min plus 3 weeks.	Quantitative	Using a tailor-made e-book with a reading habit tracking system to assess learning	Perception (of reading behaviors and strategy)
Pruet et al. (2016) (Thailand)	Grade 2, 2 urban and 2 rural primary schools	113 girls out of 213 students	Not reported	Quantitative	Using tablets in/out of the class to self-explore lessons (tablet class) for academic performance	Access (to ICT), Participation (in learning activities), perception (of ICT usage), policy (of government)
Seah and Koh (2021) (Singapore)	15 years old, a government high school	36 girls	4 weeks	Mixed	Using a mobile app in changing adolescent girls’ PA behavior, use the built-in GPS of their smartphones to track aspects of their PA such as duration, distance, pace, speed, elevation, calories burned, and route travelled.	Participation (in physical activities)
Tay (2016) (South Korea)	Secondary 1–3	420 girls (high ability, from middle/upper middle-class families)	3 years	Mixed	Using iPad in four classes to half of the school for teaching and learning activities	Access (to ICT in and out of classes), Participation (in learning activities)
(4) Computer-assisted learning						
Al-Olimat (2015) (Jordan)	Grade 10 secondary schools	72 girls	10 weeks	Quantitative	Using computer-mediated to provide corrective feedback (from T and peers) on writing	Participation (in writing activities)
Asterhan et al. (2012) (Israel)	Grade 9, a rural secondary school	38 girls out of 82 students	8 sessions (total 32 h)	Quantitative	Using a diagram-based map platform Digalo to contribute and present under teachers’ online guidance for students’ discussion.	Participation (in online discussion)
Barak and Asad (2012) (Israel)	Grade 9 in a junior high school	31 girls out of 60 students	14 weeks	Mixed	Using a computer to teach image-processing concepts	Perception (of girls’ interest in ICT/STEM)
Chen et al. (2020) (Mainland China)	Grade 2, an urban primary school	90 girls out of 178 students	1 school year	Quantitative	Using an interactive whiteboard to conduct formative assessment activities for math learning (classroom response system, collaborative-competitive gaming, feedback, etc.)	Participation (in learning activities)
Gunbatar and Karalar (2018) (Türkiye)	Grade 5–8, public middle school	39 girls out of 82 students	12 weeks	Quantitative	Teaching programming with mBlock in 12 weeks	Perception (of self-efficacy in programming)
Jeng and Liu (2016) (Taiwan)	Grade 4–6, a public primary school	209 girls out of 422 students	Not reported	Quantitative	A multimedia mental rotation test (static and interactive pentomino) to examine spatial ability for STEM	Perception (of girls’ spatial abilities)
Lau et al. (2017) (Hong Kong SAR)	Grade 6, a primary girl school	37 girls	7 weeks	Mixed	Using an online wiki-based platform in a science project for students’ online interaction to collaborate and solve a problem	Participation (in collaborative learning), Perception (of using ICT for learning)
Meishar-Tal and Shonfeld (2019) (Israel)	Mixed grades	43% girls out of 366 students	Using laptops for 1–3 year	Quantitative	Using digital devices for teaching and learning activities, including materials, feedback, and LMS.	Perception of (girls’ writing and reading style), policy (of school)
Mo et al. (2015) (Mainland China)	Mixed grades, 72 rural primary schools	Number of girls is not reported; 2741 students in total	1.5 years	Quantitative	Computer-assisted and animated resources to provide remedial math tutoring and arouse learning interest	Access to (computer and Math tutoring), participation (in Math learning activities), policy (of government)
Nusir et al. (2013) (Jordan)	Grade 1, a university model school	105 girls out of 245 students	2 sessions (30 min/ session)	Quantitative	Using interactive multimedia programs (sounds, texts, images, animations) in understanding students’ math knowledge and skills	Participation (in learning activities)
Tam et al. (2020) (Hong Kong SAR)	Grades 7 –8, a junior secondary school	411 girls	3 days	Quantitative	A local ICT training program using micro-bit and video-making	Perception (of girls’ self-efficacy, perceived value and difficulties in STEM)
Tho et al. (2015) (Hong Kong SAR)	9 secondary schools	59 girls out of 201 students	1 school year	Mixed	Innovatively used wireless Wii-mote system, Wii-mote physics freeware for learning physics concepts and recording students’ learning data.	Participation (in Physics learning), perception (of girls; interest in Physics)
Wang et al. (2015) (Taiwan/Mainland China)	Grade 11, high schools	75 girls out of 143 students	1 semester	Quantitative	Virtual physic lab/environment to provide animated results and various forms of experimental data as well as analytic tools	Access (to science inquiry skill, communication skill, process skill etc.)
Yang et al. (2013) (Mainland China)	Grade 3–4, rural public school, minority public school, migrant schools	Around 51% girls out of 6484 students	1 semester	Quantitative	Using laptops to provide computer-assisted remedial tutoring on math or Chinese language	Access (to ICT), perception (of CAL benefits boys and girls equally), policy (of government)
